# Performance Assessment of Minimum Quantity Castor-Palm Oil Mixtures in Hard-Milling Operation

**DOI:** 10.3390/ma14010198

**Published:** 2021-01-03

**Authors:** Binayak Sen, Munish Kumar Gupta, Mozammel Mia, Danil Yurievich Pimenov, Tadeusz Mikołajczyk

**Affiliations:** 1 Department of Production Engineering, National Institute of Technology, Agartala 799046, India; binayaksen3@gmail.com; 2 Key Laboratory of High Efficiency and Clean Mechanical Manufacture, Ministry of Education, School of Mechanical Engineering, Shandong University, Jinan 250100, China; munishguptanit@gmail.com or; 3 Department of Automated Mechanical Engineering, South Ural State University, Lenin Prosp. 76, 454080 Chelyabinsk, Russia; danil_u@rambler.ru; 4 Department of Mechanical Engineering, Imperial College London, South Kensington, London SW7 2AZ, UK; 5 Department of Production Engineering, University of Science and Technology Bydgoszcz, Al. Prof. S. Kaliskiego 7, 85-796 Bydgoszcz, Poland; tami@utp.edu.pl

**Keywords:** MQL, castor oil, palm oil, machining responses, Shannon’s entropy, TOPSIS

## Abstract

The necessity to progress towards sustainability has inspired modern researchers to examine the lubrication and cooling effects of vegetable oils on conventional metal cutting operations. Consequently, as an eco-friendly vegetable product, castor oil can be the right choice as Minimum quantity lubrication (MQL) base fluid. Nonetheless, the high viscosity of castor oil limits its flowability and restricts its industrial application. Conversely, palm oil possesses superior lubricity, as well as flowability characteristics. Hence, an attempt has been made to improve the lubrication behavior of castor oil. Here, six castor-palm mixtures (varying from 1:0.5–1:3) were utilized as MQL-fluid, and the values of machining responses viz. average surface roughness, specific cutting energy, and tool wear were evaluated. Furthermore, an integrated Shannon’s Entropy-based Technique for order preference by similarity to ideal solution (TOPSIS) framework was employed for selecting the most suitable volume ratio of castor-palm oil mixture. The rank provided by the TOPSIS method confirmed that 1:2 was the best volume ratio for castor-palm oil mixture. Afterward, a comparative analysis demonstrated that the best castor-palm volume fraction resulted in 8.262 and 16.146% lowering of surface roughness, 5.459 and 7.971% decrement of specific cutting energy, 2.445 and 3.155% drop in tool wear compared to that of castor and palm oil medium, respectively.

## 1. Introduction

Owing to the tremendous advantages of nickel-based superalloys, they are extensively utilized in the aerospace, nuclear, and aviation industries. However, the machinability of these alloys is very poor because of their extreme mechanical properties [1]. On top of the high tensile and share strength of these superalloys, superior tensile and share strength are conferred, which eventually escalates the work hardening phenomenon [2,3]. At shallow cutting speed and feed rate, tool damage is also a significant spectacle during machining of these alloys. Due to the tool-work interaction, high heat is generated on the cutting zone; thus, small chips were getting welded on the rake and flank faces of the cutting tool and produced a built-up edge. Thus, poor work-surface morphology and significant tool damage can be observed [2]. Eyeing these challenges, the application of lubricants is a prominent choice to moderate the frictional effect in the machining zone [4,5]. However, in conventional lubrication strategy, gallons of lubricants are being used in the industries and disposed of in the environment, which is perhaps harmful for the eco-system and human health [6]. Thus, MQL technology is the minimum quantity lubrication strategy considered to be the most convenient solution to meet such imposing trade-off challenges [3].

Over the past decade, extensive research has been conducted by various researchers on vegetable oil-based lubricants. For instance, Belluco and De Chiffre (2004) used five different vegetable oils as a lubricant during machining of AISI 316 L, where HSS-CO was used as tool material. During experimentation, they measured cutting forces, tool life, and chip length. Finally, the researchers found up to 177% increment in tool life [7]. To observe the lubricating ability of vegetable oil, Ojolo et al. (2008) executed a machining operation using groundnut, palm, kernel, shear butter, and coconut oil. Aluminium, mild steel, and copper were machined using tungsten carbide tools. To this end, the authors conferred that the palm-kernel and ground-nut oil performs better for minimizing the force exerted by the tool on the work [8]. About a decade ago, Khan et al. (2009) conducted a machining operation on AISI 9310 material using an uncoated carbide tool. During machining, vegetable oils were sprayed into the tool-work interface using an MQL device. Furthermore, the authors compared the machining responses of the MQL medium with the dry and wet medium. The results of this study depicted that MQL medium is highly beneficial while reducing the surface roughness, tool-work temperature and cutting forces [9]. In the same year, Xavior and Adithan (2009) investigate the lubricating ability of emulsion, coconut oil, and net cutting oil during the machining operation. As an output, the authors observed the said lubricants’ effect on surface morphology and tool wear profile. The outcomes of this research depicted that coconut oil has a significant impact on improving the texture of the machined surface and minimizing the tool wear morphology, where the effect of net cutting oil and emulsion on the machining responses were insignificant [10]. Kuram et al. (2010) performed a drilling operation of AISI 304 steel using the HSS-E tool. The authors experimentally observed the influences of vegetable oil-based metal cutting fluids on the machining outputs, surface roughness, and thrust force. The results of this particular study manifested that at a spindle speed of 720 rpm commercial oil performs better as compared to vegetable oils [11]. Sharif et al. (2013) discovered the lubricity of palm oil and fatty-alcohol during milling of AISI 420. Throughout this study, the authors observed a rapid progression of tool wear in a dry and flooded condition. However, for fatty alcohol and palm oil-based lubricating conditions, the progression of the wear was steady [12].

During machining of GH4169, Wang et al. (2016) observed the lubricating ability of various vegetable oils. Based on experimental results, the following ranks were established: maize oil < rapeseed oil < soybean oil < sunflower oil < peanut oil < palm oil < castor oil [13]. However, Li et al. (2016) observed that castor oil is not a proper choice as a lubricant/coolant. Their high viscosity value restricts flowability characteristics. Thus, castor oil couldn’t be used as a metalworking fluid. In contrast, palm oil possesses very good polarity and flowability as well [14]. Contemplating these findings of the previous researchers, an experimental endeavor was attempted to understand the lubricating behavior of castor-palm oil mixtures under a machining environment. Additionally, this study also determined the optimal castor-palm volume ratio for MQL milling of Inconel 690. The TOPSIS with regard to weights derived from Shannon’s entropy has been used to determine the optimum castor-palm volume ratio in the MQL milling endeavor. In view of the above literature, this effort, thus, prolongs the laid in the literature further to examine the lubricating performance of mixed vegetable oils under the machining environment.

## 2. Materials and Methods

### 2.1. Machining Operation

The CNC-milling operation was conducted on nickel-based super alloys called Inconel 690 (150 × 80 × 10 mm). Uncoated carbide end-mills were used during machining operations for their tremendous capacity to withstand high temperatures. The specifications of the used end-mill are shown in Table 1. Besides, the chemical composition of castor and palm oil is presented in Table 2 and Table 3. The mixtures of castor-palm oil (varying from 1:0.5–1:3) were sprayed into the tool-work interfaces using an MQL supply system (KRS, India). The specification of the MQL device is shown in Table 4. Vitamin C tablets were added to the prepared mixtures to improve the oxidation resistance of used vegetable oils. After an extensive literature review, the range of machining and MQL parameters were selected [15,16], which are shown in Table 5. The experimental strategy of the present study is detailed in Table 6.

**Figure 1:**
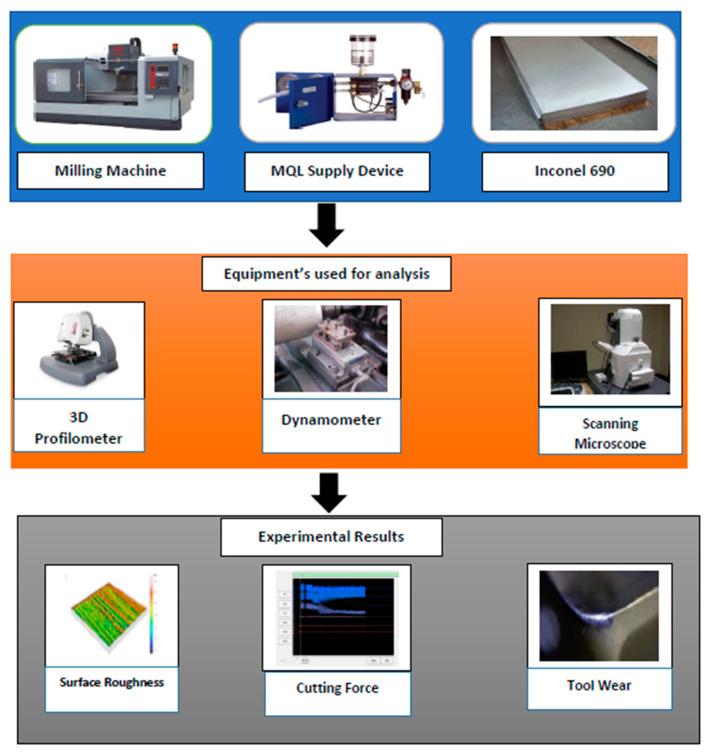
Framework of the present study.

Finally, the machining responses were measured for different castor-palm mixtures. Each experiment was repeated three times, and the average response values were considered. The roughness of the machined surfaces (*R_a_*) was calculated using a 3D Profilometer (Taylor Hobson, India). In this study, surface roughness was calculated from five different points, and the mean of these values was considered as average surface roughness. A Kistler-9257-BA dynamometer (Kistler Instrument, India) was attached to the workpiece. LabVIEW^®^ software was used for capturing the cutting force data-base. Here, an amplifier was used to amplify the signals received from the dynamometer at a sampling frequency of 7000 Hz. The machining forces subjected by the workpiece were divided into three components. The resultant of these forces were calculated by applying Equation (1). Subsequently, the specific energy was calculated by using Equation (2). Finally, a scanning electron microscope (SEM) (Carl ZEISS, India) was utilized to observe the flank wear (*V_B_*) profile of the end-mill. Figure 1 shows the overall framework of the present study.

(1) $$\mathrm{Resultant}\;\mathrm{cutting}\;\mathrm{force}\;\left(F_{r}\right)=\sqrt{F_{x}^{2}+F_{y}^{2}+F_{z}^{2}}$$

(2) $$\mathrm{Specific}\;\mathrm{cutting}\;\mathrm{energy}\;(E_{sp})\;=\;\frac{\mathrm{Resultant}\;\mathrm{Cutting}\;\mathrm{Force}}{\mathrm{Feed}\;\mathrm{rate}\;\times \;\mathrm{Depth}\;\mathrm{of}\;\mathrm{Cut}}\;(N/{\mathrm{mm}}^{2})$$

### 2.2. TOPSIS Based Ranking Strategy with Entropy Weight

Hwang and Yoon developed TOPSIS in 1981 [19]. Since then, this MCDM method has been widely applied in various engineering sectors and emerged as the most prevalent decision-making method. TOPSIS uses the knowledge of a compromise solution for ranking the alternatives. Actually, TOPSIS helps the researchers to rank the alternatives by deriving compromise indexes based on the distances of the alternatives from a positive ideal solution (PIS) and negative ideal solution (NIS). The mathematical explanation of the decision and weight information is described below:

- $A\;\left(coolection\;of\;alternatives\right)=\left\{A_{i}:i\in ℐ\right\}$ where ℐ={1,2,…,m}
- $C\;\left(Set\;of\;Criteria\right)=\left\{C_{j}:j\in T\right\}$ where T={1,2,…,n}
- The weight vector: $\left(w_{1},\;w_{2},\ldots ,w_{n}\right)$ where, $w_{j}\;\geq 0\;\forall \;j$ and $\sum_{j=1}^{n}w_{j}$ = 1
- The decision matrix (D) is shown in Equation (3):
  (3) $$\;C_{1}\;C_{2}\;\ldots \;C_{n}\;D\;=\;\begin{array}{c} A_{1}\\ A_{2}\\ \vdots \\ A_{m} \end{array}\left(\begin{array}{cccc} a_{11} & a_{12} & \cdots & a_{1n}\\ a_{21} & a_{22} & \cdots & a_{2n}\\ \vdots & \vdots & \cdots & \vdots \\ a_{m1} & a_{m2} & \cdots & a_{mn} \end{array}\right)$$
  where $a_{ij}$ signifies the performances of the alternatives against the criteria *Cj*. The raking method of TOPSIS is described below:
  **Step 1**: Normalization of the D using Equation (4):
  (4) $$r_{ij}=\;\frac{a_{ij}}{\sqrt{\sum_{i=1}^{m}a_{ij}^{2}}}$$
  **Step 2**: An essential component of MCDM methods is determining the weight of each criterion. Previous literature has suggested different techniques to generate criteria weights. They can be categorized into the following groups:
  (a) ***Subjective approach*:** In this approach, the weight of any criteria is defined based on the decision maker’s preferences.
  (b) ***Objective approach:*** in this approach, weight is directly calculated from the decision matrix.
  In the present study, an entropy objective approach has been applied to determine the weightage of the machining responses. The idea of entropy is utilized extensively to calculate the uncertainty related to the information [20,21,22]. The entropy-based weight can be determined from the normalized decision matrix by applying the following steps:
- Calculation of entropy by applying Equation (5):
  (5) $$E_{j}=\;-\frac{1}{\ln n}\overset{m}{\underset{ij}{\sum }}r_{ij}\ln r_{ij}\;\forall \;j$$
- The diversification strength of the information provided by the outcome under criterion j is calculated by Equation (6):
  (6) $$\;d_{j}=1-E_{j}\;\forall \;j$$
- When the decision-maker has no extra preference information over the criteria, the *principle of insufficient reason* infers the best weights of the criteria are given in Equation (7):
  (7) $$w_{j}=\frac{d_{j}}{\sum_{j=1}^{n}d_{j}}\;\forall \;j$$

The weighted normalized decision matrix $U=\;{\left(u_{ij}\right)}_{m\times n}$ is obtained by using Equation (8):

(8) $$u_{ij}=\;w_{j}\;\times \;r_{ij},\;i\;\in \;𝚥,\;j\;\in \;𝚥\;$$

**Step 3**: Calculation of PIS and NIS (Equations (9) and (10))

(9) $$PIS=\left(u_{1}^{+},\;u_{2}^{+},\;\ldots \;u_{n}^{+}\right),\;\mathrm{where}\;u_{j}^{+}=\;max_{i}\;u_{ij}$$

(10) $$NIS=\left(u_{1}^{-},\;u_{2}^{-},\;\ldots \;u_{n}^{-}\right),\;\mathrm{where}\;u_{j}^{-}=\;min_{i}\;u_{ij}$$

**Step 4:** The separation measure for the alternative $A_{i}$ from PIS is computed by using Equation (11):

(11) $$SM_{i}^{+}=\;\sqrt{\sum_{j=1}^{n}{\left(u_{ij}-u_{i}^{+}\right)}^{2}}$$

Correspondingly, the separation measure from NIS is calculated by using Equation (12):

(12) $$SM_{i}^{-}=\;\sqrt{\overset{n}{\underset{j=1}{\sum }}{\left(u_{ij}-u_{i}^{-}\right)}^{2}}$$

**Step 5**: Computation of the relative closeness coefficient (*RC_i_*) to the ideal solutions by applying Equation (13):

(13) $$RC_{i}=\;\frac{SM_{i}^{-}}{SM_{i}^{+}+\;SM_{i}^{-}}$$

**Step 6**: Finally, alternatives are ranked with the principle that the best alternative has a maximum *RC_i_* value.

## 3. Results and Discussion

### 3.1. Variation of Machining Responses with the Castor-Palm Ratio

The surface roughness is the prime index of the geometrical quality of the workpiece. It is determined by the scratching of tool material on the surface of the workpiece. A lower surface roughness value achieves better fatigue, corrosion, and abrasion resistance of the workpiece and higher cooperation accuracy [23,24]. Here, the values of *R_a_* for different castor-palm mixtures were as follows: castor-palm oil mixture (1:0.5), 0.375 µm; castor-palm oil mixture (1:1), 0.341 µm; castor-palm oil mixture (1:1.5), 0.389 µm; castor-palm oil mixture (1:2), 0.322 µm; castor-palm oil mixture (1:2.5), 0.361 µm; castor-palm oil mixture (1:3), 0.421 µm.

Specific cutting energy implies the energy needed to remove a unit volume of material from the workpiece. In actuality, this parameter depicts the energy consumed during the machining process. Now and then, it was used as an efficiency indicator of machining processes [25]. From the experimental analysis, it was evident that the specific cutting energy of castor oil (1:0) was the largest (0.3991 N/mm^2^), whereas the specific cutting energy of the castor-palm oil mixture decreases to varying degrees relative to the mixture volume of castor and palm oil. The specific cutting energy of castor-palm (1:2.5) was least (0.3526 N/mm^2^). 

**Figure 2:**
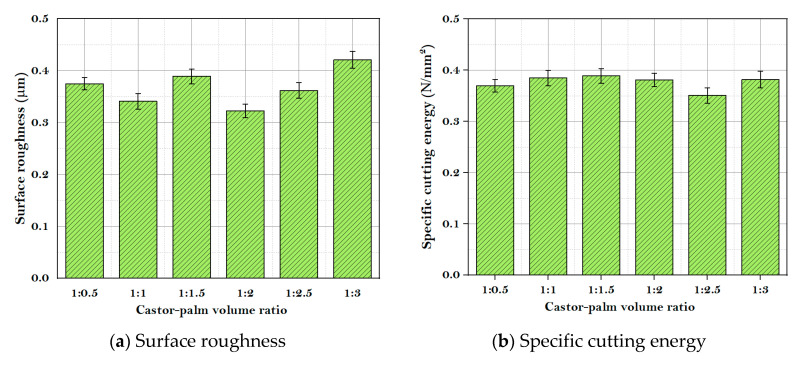

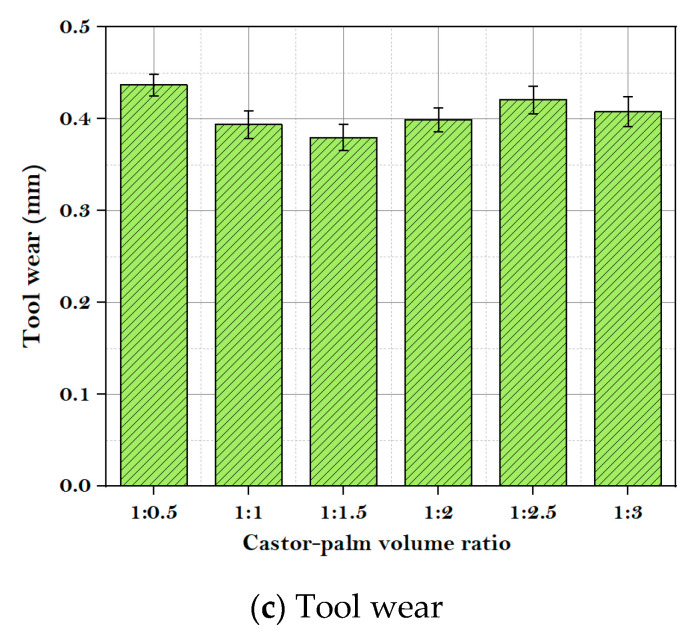
(**a**–**c**): Machining performances under different volume fraction of castor-palm oil.

Finally, the tool wear refers to the ongoing failure of the cutting tool due to the continuous friction between the tool and workpiece [25]. The experimental outcomes revealed that the application of castor-palm oil mixture is an effective tactic to minimize the tool wear rate also. The comprehensive values of tool wear for oil mixtures were as follows: castor-palm oil mixture (1:0.5), 0.436 mm; castor-palm oil mixture (1:1), 0.394 mm; castor-palm oil mixture (1:1.5), 0.381 mm; castor-palm oil mixture (1:2), 0.399 mm; castor-palm oil mixture (1:2.5), 0.412 mm; castor-palm oil mixture (1:3), 0.406 mm. Figure 2a–c demonstrates the values of machining performances under different volume fractions of castor-palm oil.

### 3.2. Result of the Proposed MCDM Model

Experimental results of this study demonstrated that with the changing volume fraction of castor-palm oil mixture, the machining performances had changed differently. Hence, the ideal castor-palm volume ratio selection is a challenging task for the researchers. Nevertheless, the MCDM techniques have an excellent reputation in the decision-making domain [25,26,27,28,29]. Thus, the most common MCDM method—TOPSIS—was implemented to choose the best alternative. Furthermore, TOPSIS requires an effective method for weight calculation. Therefore, Shannon’s entropy method was also utilized to compute the weights of each machining response.

**Figure 3:**
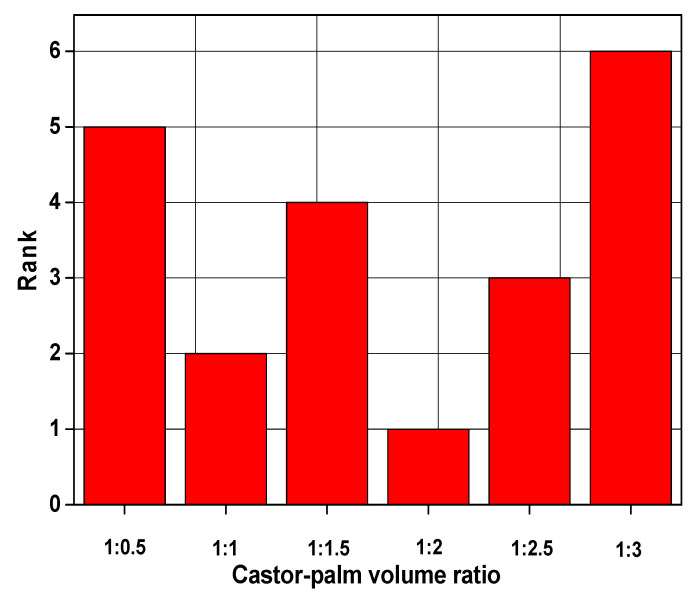
Ranking of alternatives by TOPSIS method.

In this study, 6 × 3 decision matrix was prepared based on the experimental results, and it is denoted as D = ${\left(a_{ij}\right)}_{6\times 3}$ where *m* = 1, 2,……,6 is the number of the castor-palm mixture and n=1,2,3 is the number of performance parameters. We consider each of the experimental runs as an alternative and corresponding output as the performance measuring criteria. It is to be noted that all the performance measuring criteria belong to the cost category, i.e., minimization type. To find the best value of castor-palm oil volume ratio, minimization characteristics for *R_a_*, *E_sp_*, and *V_B_* are considered. The calculated weight factor of *R_a_*, *E_sp_*, and *V_B_* was found to be 0.3329, 0.3336, and 0.3335, respectively. Furthermore, the most suitable volume ratio of castor-palm oil mixture was calculated by the TOPSIS approach. From Figure 3, it is apparent that the 4th run obtains the first rank.

### 3.3. Molecular Structure of Green Mixtures

Castor oil is mainly composed of ricinoleic ester (90%), dilinolein, triolein, stearin, and fatty acids [30]. After mixing palm oil in castor oil, the proportion of ricinoleic ester was reduced, and the proportion of saturated (palmitic acid), mono-saturated (oleic acid), and polyunsaturated (Linoleic acid) fatty acid were increased. Actually, the lubrication effect of the mixture during milling is contributed due to the hydroxyl (-OH) groups and polar molecules (-COOH, -COO^−^) presented in the vegetable oil. Due to strong attraction and high adsorption energy, the mixture of castor-palm oil is easily adsorbed on the metal surface, thus developing a strong lubrication film. As the proportion of castor oil decreased in the mixture, the -OH groups in the mixture became nearly equal to that of saturated (palmitic acid), mono-saturated (oleic acid), and polyunsaturated (Linoleic acid) fatty acid. Due to the high amount of cutting temperature, esterification took place between the -OH groups and fatty acids, hence offering efficient lubrication performance. Once the proportion of castor oil in the mixture was minimal (1:3), insufficient ricinoleic acid molecules could persuade esterification. Due to high palmitic, oleic, and linoleic acid content on the mixture, the oil film density reduced and could not provide desirable lubrication performance.

### 3.4. Viscosity of Green Mixtures

**Figure 4:**
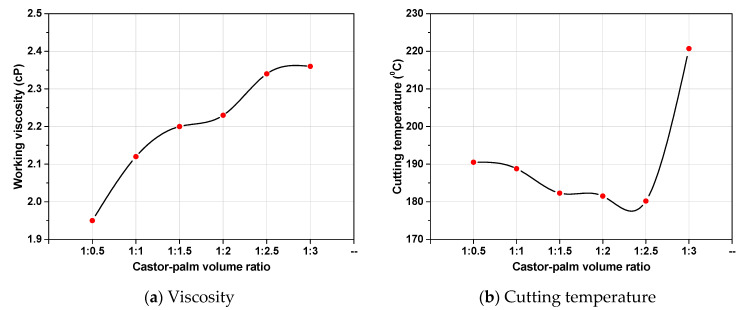
(**a**,**b**): Variation of viscosity and cutting temperature with castor-palm volume ratio.

Viscosity has a great impact on lubrication performance. It is the ratio of oiliness and mobility of the lubricants [31]. The high value of viscosity offered strong lubrication film. However, viscosity declined with an increment in temperature since the molecular distance of vegetable oil is negligible. In actuality, once the temperature of the vegetable oil increased, correspondingly, molecular kinetic energy, flow between the molecules, the power of the liquid increased, and dynamic viscosity reduced. From Figure 4a, it was evident that the castor-palm oil mixture (1:3) had the highest viscosity and is assumed to have the best lubricity. Although in actuality, it possesses poor lubrication capability according to the investigation of *Ra*, *E_sp_*, and *V_B_*. This outcome may be associated with the low-heat transfer capacity of the castor-palm mixture. From Figure 4b, it was also evident that the castor-palm oil mixture (1:3) showed the maximum cutting temperature. As the castor-palm volume ratio varied from 1:0.5–1:2.5, the cutting temperature was comparatively low. After 1:2.5, the cutting temperature rose significantly. In actuality, lubrication film’s desorption phenomenon took place under a higher temperature; thus, lubricants can’t provide effective lubrication [32,33].

### 3.5. A Comparative Study

The machining performances obtained from the best castor-palm volume fraction (1:2) have been compared with the machining performances of castor and palm oil. A comparison that MQL milling with castor-palm volume fraction (1:2) demonstrated much better machining responses than other medium (Table 7). In fact, the best castor-palm volume fraction resulted in 8.262 and 16.146% lowering of surface roughness, 5.459 and 7.971% decrement of specific cutting energy, 2.445 and 3.155% drop in tool wear compared to that of castor and palm oil medium, respectively.

**Figure 5:**
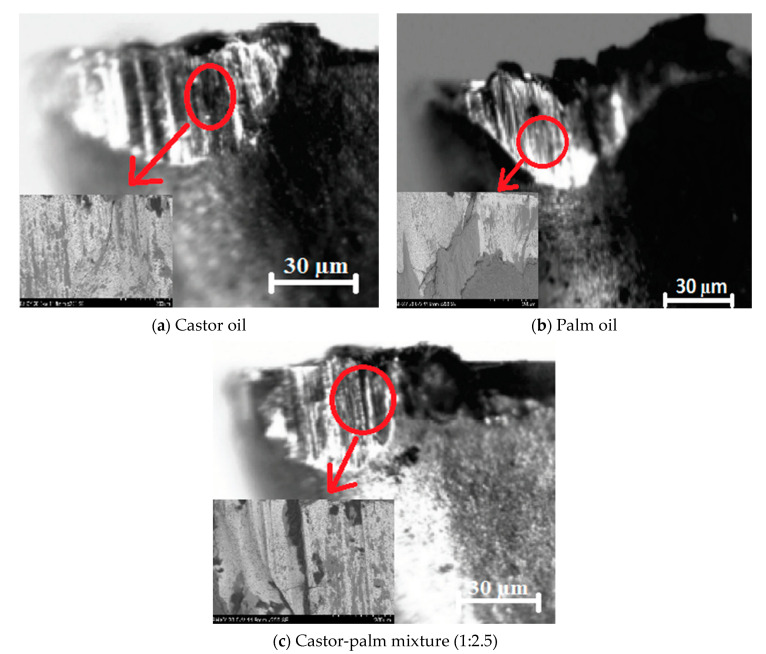
(**a**–**c**): Thermal cracks in the tool during machining of Inconel 690.

Furthermore, OM and SEM have been utilized to observe the flank wear mechanism for three cooling conditions: castor oil, palm oil, and castor-palm oil (1:2) mixture. From Figure 5a–c, it was depicted that thermal fatigue was the primary cause of tool wear in all cooling conditions, which was related to the gradual deterioration of cutting edges. Along with thermal cracks, material adhesion and worn out of tool coating were also observed. The adherent material in the tool indicated an adhesive wear mechanism. Indeed, vegetable oils did not alter the tool wear mechanism but significantly reduced the tool wear rate. In summary, the present study showed that the optimum castor-palm oil mixture has exceptional ability to improve the machining responses for milling of superalloys. The optimum mixture of castor and palm oil showed better results as compared to castor and palm oil. In addition, the vegetable oils, which were used in the present study, were chemical/additive-free, which might contribute to achieving a sustainable manufacturing environment and also addresses the serious issues of environmental pollution.

## 4. Conclusions

In this experimental study, castor-palm mixtures were utilized as MQL base fluid in the milling of Inconel 690 to investigate the effects of castor-palm mixtures on the milling performance parameters (*R_a_*, *E_sp_*, and *V_B_*). The authors subsequently applied Shannon’s entropy coupled TOPSIS technique for selecting the preferable volume ratio of castor-palm oil mixtures. To this end, a comparative study was performed to check the supremacy of the proposed green lubricant in the MQL milling environment. Thus, the following conclusions have been made:

- With the increasing volume fraction of palm oil, the value of surface roughness was decreased first, then upsurged. The minimum value of surface roughness was achieved at the castor-palm volume fraction (1:2). Conversely, the maximum value was achieved at 1:3. The minimum specific energy was attained when the castor-palm volume mixture is 1:2.5. Furthermore, the minimum tool wear was founded at 1:1.5.
- To improve the machining economy and efficiency, the selection of proper lubricant is a crucial concern. In this context, Shannon’s entropy-based TOPSIS approach was applied to determine the best castor-palm volume ratio. The ranking of Shannon’s entropy-based TOPSIS conferred that Castor-palm volume fraction (1:2) is best for minimizing machining responses.

Finally, a comparative study shows that MQL assisted milling with a castor-palm volume ratio (1:2) confirmed much better machining responses than castor and palm oil medium. Furthermore, thermal fatigue was the primary cause of tool wear for castor oil, palm oil, and castor-palm oil (1:2) condition.

## Figures and Tables

**Table 1:** Specifications of End-mill.

Specification	Description
Base metal	Fine-grained cemented carbide
Diameter	6 mm
Flutes	4
Length	83 mm
Rake angle	6°
Helix angle	30°
Clearance angle	15°
Grain size	1 µm

**Table 2:** Chemical composition of castor oil [17].

Acid Name	Average Range (%)
Ricinoleic acid	84.5–94
Oleic acid	3–7
Linoleic acid	1.5–6
α-Linolenic acid	0.4–1
Stearic acid	0.4–1
Palmitic acid	0.4–1
Dihydroxystearic acid	0.25–0.6
Others	0.25–0.6

**Table 3:** Chemical composition of palm oil [18].

Acid Name	Average Range (%)
Myristic acid	1
Palmitic acid	43.5
Stearic acid	4.3
Oleic acid	36.6
Linoleic acid	9.1
Others	5.5

**Table 4:** Technical specifications of MQL system.

Specification	Description
Pumping elements	2 (two)
Capacity of reservoir	5 L
Operating source	Compressed air
Type of spray	Mist spray
Functional temperature	−25 °C–75 °C
Air pressure	5–15 Bar
Flow rate	0–300 mL/h
Kinematic Viscosity	25–150 Cst.

**Table 5:** MQL assisted milling conditions.

Parameters	Values
Cutting speed	140 m/min
Feed	0.2 mm/tooth
Depth-of-cut	1.0 mm
Flow rate of lubricant	120 mL/h
Nozzle distance	30 mm
Nozzle angle	15°
Pressure	0.8 MPa

**Table 6:** Experimental strategy with different Castor-Palm oil volume ratio.

Experiment No.	Castor-Palm Oil Volume Ratio	Lubrication Condition
1	1:0.5	Minimum Quantity Lubrication
2	1:1	
3	1:1.5	
4	1:2	
5	1:2.5	
6	1:3	

**Table 7:** Machining performances in different cooling condition.

Machining Performances	Castor Oil	Standard Deviations	Palm Oil	Standard Deviations	Castor-Palm Mixture (1:2)	Standard Deviations
*R_a_* (µm)	0.351	0.005	0.384	0.042	0.322	0.074
*E_sp_* (N/mm^2^)	0.403	0.007	0.414	0.064	0.381	0.043
*V_B_* (mm)	0.409	0.004	0.412	0.012	0.399	0.021

## Data Availability

Not applicable.

## Nomenclature

CNC	Computer numerical control
MQL	Minimum quantity lubrication
SEM	Scanning electron microscope
OM	Optical microscope
*R_a_*	Surface roughness
*F_r_*	Resultant cutting force
*E_sp_*	Specific energy
*V_B_*	Tool wear
MCDM	Multi-criteria decision-making method
TOPSIS	Technique for order preference by similarity to ideal solution
PIS	Positive ideal solution
NIS	Negative ideal solution
*RC_i_*	Relative closeness coefficient
